# Environmental and occupational respiratory diseases – 1043. Effects of socio-economic status, life style patterns and demographic factors

**DOI:** 10.1186/1939-4551-6-S1-P42

**Published:** 2013-04-23

**Authors:** Monika Bhuker, Satwanti Kapoor

**Affiliations:** 1 Anthropology, Delhi University, Delhi, India

## Background

Asthma is increasing among adults in Delhi and its fundamental cause is not known yet. Identifying the potential risk factors may be helpful to understand its cause.

## Methods

A community based study of 50 cases and 50 controls ranging in age from 18 to 50 years was undertaken in Delhi region of India. The cases were collected from Dr. B.R Ambedkar Hospital, Rohini. The controls were chosen from the same demographic region. Statistical package for social studies (SPSS ver.17) was used to find statistical significance.

## Results

In clinical cases of asthma, poor sanitation and water supply (p<0.01), low education level (p<0.01), low income (p<0.01), positive family history and low physical activity (p<0.01) were found to be associated with asthma. We also found that low education level (p<0.001,6.75 times), low family income (p<0.001,9.11 times), low personal income (p<0.001,12.5times), poor sanitation (p<0.01,29.9 times) and high sensitivity to allergy (p<0.001,17.07 times), positive family history (p<0.01,2.78 times) were likely to increase the risk of asthma in the population under study. Our study also suggested that house wives are 4.80 times (p<0.05) and inactive individuals are 9.54(p<0.001) times more at risk of developing asthma.

## Conclusions

This study indicates that low socio-economic status, positive family history of asthma, higher sensitivity to allergy, inactive life style and indoor environmental risk factors are the potential risk factors for asthma in adult Brahmin population in Delhi, India.

